# SPG302 Reverses Synaptic and Cognitive Deficits Without Altering Amyloid or Tau Pathology in a Transgenic Model of Alzheimer’s Disease

**DOI:** 10.1007/s13311-021-01143-1

**Published:** 2021-11-04

**Authors:** Laura Trujillo-Estrada, Peter W. Vanderklish, Marie Minh Thu Nguyen, Run Rong Kuang, Caroline Nguyen, Eric Huynh, Celia da Cunha, Dominic Ibarra Javonillo, Stefania Forner, Alessandra C. Martini, Stella T. Sarraf, Vincent F. Simmon, David Baglietto-Vargas, Frank M. LaFerla

**Affiliations:** 1 Institute for Memory Impairments and Neurological Disorders, University of California, Irvine, CA 92697 USA; 2 Department of Neurobiology and Behavior, University of California, Irvine, CA 92697-1450 USA; 3 Spinogenix Inc, 10210 Campus Point Drive, Suite 150, San Diego, CA 92121 USA; 4 Departamento Biología Celular, Genetica y Fisiologia, Instituto de Investigacion Biomedica de Malaga-IBIMA, Facultad de Ciencias, Universidad de Malaga, Malaga, Spain; 5 Centro de Investigacion Biomedica en Red sobre Enfermedades Neurodegenerativas (CIBERNED), Madrid, Spain

**Keywords:** Alzheimer’s disease, Dendritic spines, Synaptic deficits, 3xTg-AD mice, SPG302, Synaptic markers

## Abstract

Alzheimer’s disease (AD) is conceptualized as a synaptic failure disorder in which loss of glutamatergic synapses is a major driver of cognitive decline. Thus, novel therapeutic strategies aimed at regenerating synapses may represent a promising approach to mitigate cognitive deficits in AD patients. At present, no disease-modifying drugs exist for AD, and approved therapies are palliative at best, lacking in the ability to reverse the synaptic failure. Here, we tested the efficacy of a novel synaptogenic small molecule, SPG302 — a 3rd-generation benzothiazole derivative that increases the density of axospinous glutamatergic synapses — in 3xTg-AD mice. Daily dosing of 3xTg-AD mice with SPG302 at 3 and 30 mg/kg (i.p.) for 4 weeks restored hippocampal synaptic density and improved cognitive function in hippocampal-dependent tasks. Mushroom and stubby spine profiles were increased by SPG302, and associated with enhanced expression of key postsynaptic proteins — including postsynaptic density protein 95 (PSD95), drebrin, and amino-3-hydroxy-5-methyl-4-isoxazolepropionic acid receptor (AMPAR) — and increased colocalization of PSD95 with synaptophysin. Notably, SPG302 proved efficacious in this model without modifying Aβ and tau pathology. Thus, our study provides preclinical support for the idea that compounds capable of restoring synaptic density offer a viable strategy to reverse cognitive decline in AD.

**Supplementary Information:**

The online version contains supplementary material available at 10.1007/s13311-021-01143-1.

## Introduction

Alzheimer’s disease (AD) is a progressive neurodegenerative disorder and the leading cause of dementia world-wide, estimated to afflict 50 million people by 2050. In the USA, the estimated total expenses for AD in 2020 is $305 billion, with the cost expected to increase to more than $1 trillion by 2050 [1]. Despite its prevalence, and greater than century long recognition as a clinical entity, disease-modifying therapies remain elusive [2]. In the last 20 years, over 150 clinical stage drugs have failed, dominated recently by a string of failures among therapies targeting amyloid beta (Aβ), and the last FDA approval was in 2003 [3]. Current therapies for AD, such as acetyl cholinesterase (AChE) inhibitors and N-methyl-D-aspartate receptor (NMDAR) antagonists, afford only temporary symptomatic relief without modifying disease progression long term. Prompted by recent clinical failures and new insights into the etiology of AD, a large pipeline of candidate therapeutics has emerged that addresses diverse aspects of AD pathogenesis at the molecular level [3]. While debate continues about which therapeutic focus points offer the best chance for disease modification, what is not in question is that for any therapeutic to be successful it must address one of the most functionally significant consequences of AD pathogenesis: synaptic failure [4].

AD is the prototypical synaptopathy, the best characterized member of an etiologically diverse set of neurodegenerative and other conditions in which the dysfunction and loss of synapses, primarily excitatory glutamatergic contacts, gives rise to deficits in cognition and other functions [4–8]. In AD, a progressive loss of glutamatergic synapses uncouples cortical and limbic circuits involved in learning, memory, and cognition [5, 6, 9–11]. Early histological studies that quantified numbers of synapses in biopsied [5, 12] or postmortem [5, 6, 13–18] brain tissue from AD patients revealed large reductions in synaptic density (15–45%) in the hippocampus, entorhinal cortex, and in several neocortical and subcortical regions. Collectively, these data (reviewed in [15, 19, 20]) indicate that synapse loss occurs early in AD and progresses in a way that correlates strongly with cognitive decline. Importantly, morphometric and statistical considerations dictate that most of the synapses lost are glutamatergic contacts in which the postsynaptic elements are dendritic spines, the highly specialized, actin-rich protrusions of dendritic membrane that comprise the postsynaptic element in ~ 90% of glutamatergic contacts [21]. The loss of axospinous contacts is particularly critical as they are loci of plasticities such as long-term potentiation (LTP) and depression (LTD) that alter the function of corticolimbic circuits in support of memory formation [22]. Correlations between the loss of dendritic spine synapses and cognitive decline are supported by work in mouse models [20, 23–28], by studies of the spine actin regulatory proteins spinophilin [29, 30], cofilin [31], and drebrin [32], as well as 3D electron microscopy in postmortem AD brain [33, 34]. The recent application of PET imaging of synaptic density to AD, using the SV2A ligand UCB-J, has validated that synapse loss occurs in vivo and correlates with cognitive status, while also providing a clinically translatable biomarker of synapse loss [35, 36]. Collectively, these and other observations raise the prospect that the regeneration of synapses, not merely the slowing of underlying molecular processes that lead to their dysfunction and loss, should be an explicit goal of AD drug development [5]. Observations that cognition is spared in patients who, despite robust Aβ and neurofibrillary tangle pathology, have normal densities of dendritic spines (thought to be a basis of “cognitive reserve”) support this notion [37].

Among a relatively short list of strategies to induce synaptogenesis in vivo [38], the modulation of cellular pathways that regulate the actin cytoskeleton is a particularly attractive approach in the context of AD where there is evidence of early dysregulation contributing to synaptic failure. Dendritic spine structure is determined in large part by highly branched assemblies of filamentous actin (F-actin) [39–41], dynamics in which contribute to spine shape changes during LTP and other plasticities [42–44]. Importantly, the assembly of branched F-actin networks is a necessary initial step in spine formation [45, 46]. The organization of F-actin in spines is regulated by an ecosystem of actin-binding proteins (ABPs) [41, 44, 47], the dysregulation of many of which has been shown to result in abnormalities in spine shape and cognitive function [44], including in AD. For example, expression levels of drebrin, a key postsynaptic ABP involved in the clustering and stabilization of F-actin in spines [47] and spine maturation, are reduced in the hippocampus and neocortex of AD patients, and in the superior temporal cortex of those with prodromal mild cognitive impairment, correlating with cognitive status [47–49]. In addition, activity of the F-actin severing protein, cofilin, is upregulated in AD. Dephosphorylation of cofilin activates its actin-severing function, which leads to disassembly of spine F-actin, spine retraction [31, 50], reduced LTP and spine density, and enhanced LTD [51–53]. Conversely, phosphorylation of cofilin inhibits this activity and is associated with spine enlargements underlying LTP consolidation [53]. Large reductions in phospho-cofilin are seen early in AD brain and in mouse models at a young age, contributing to synaptic failure and the appearance of characteristic cofilin/actin rods [31, 54, 55]. Such observations point to a compromised synaptic F-actin cytoskeletal network that contributes to synaptic failure and a reduced ability to form new synapses.

SPG302 is a 3rd-generation member of a novel class of pegylated benzothiazole derivatives that target an undisclosed regulator of the F-actin-based cytoskeleton and increase dendritic spine density in vitro and in vivo. Spines induced by SPG compounds comprise new synapses and exhibit the normal distribution of spine shapes found in wild type mouse brain, without inducing supraphysiological densities of synapses. Previous studies from our laboratory demonstrated that triple transgenic (3xTg-AD) AD mice develop cognitive and synaptic deficits at an early stage of the disease, well before the appearance of plaques and tangles [56]. These cognitive and synaptic alterations were associated with changes in AMPA receptor signaling, which is compromised early in the disease course in AD and ABPs, including drebrin [57]. In the present study, we tested the effects of SPG302 on cognition, synaptic density, key synaptic proteins, and the levels of Aβ and phosphorylated Tau in the 3xTg-AD mouse. In 6-month old 3xTg-AD mice, SPG302 was able to rescue deficits in cognition in hippocampal-dependent tasks and reverse substantial deficits in dendritic spines to densities that were not statistically different from wild type controls. This improvement in behavior was associated with increases in the expression of postsynaptic proteins (such as GluA1 and drebrin) and the co-localization of pre- and post-synaptic markers. Notably, these synaptic and cognitive enhancements were not associated with any changes in AD-like pathology as measured by hippocampal levels of Aβ and phosphorylated Tau. These data advance SPG302 as a novel and promising therapeutic for AD, and support the concept that promoting synapse regeneration through cytoskeletal acting small molecules may have broad utility in the synaptopathies.

## Materials and Methods

### Transgenic Mice and Treatment Protocol

Female homozygous 6-month-old 3xTg-AD and WT mice were used in the current study. The characterization of the 3xTg-AD mice has been described previously [56]. In brief, two independent transgenes encoding human APP_swe_ and the human Tau_P301L_ (both under the control of the mouse Thy1.2 regulatory element) were co-microinjected into single-cell embryos harvested from homozygous mutant PS1_M146V_ knockin (PS1-KI) mice [56].

Two different doses of the drug SPG302 were used: 3 mg/kg and 30 mg/kg. 3xTg-AD and WT mice were randomly divided into three groups (*n* = 8–11): WT vehicle (*n* = 11), WT SPG302 3 mg/kg (*n* = 11), WT SPG302 30 mg/kg (*n* = 10), 3 × vehicle (*n* = 10), 3 × SPG302 3 mg/kg (*n* = 10), 3 × SPG302 30 mg/kg (*n* = 10). SPG302 (diluted in 5% dimethyl sulfoxide DMSO) was administered by intraperitoneal injection once daily for 4 weeks. Animals treated with vehicle (5% DMSO in phosphate buffered saline PBS) were used as a control group. SPG302 was supplied by Spinogenix, which provided funding for this study. All animal procedures were performed in accordance with NIH, University of California guidelines and Use Committee at the University of California, Irvine.

### Behavioral Testing

#### Morris Water Maze

Hidden Morris water maze (MWM) tests were conducted as described [58]. A circular aluminum tank (1.5-m diameter) was used for MWM experiments. Mice were trained to swim to a 14-cm-diameter circular Plexiglas platform submerged 1.5 cm beneath the surface of the water and invisible to the mice while swimming. The platform was located in a fixed position, equidistant from the center and the wall of the tank. Mice were subjected to four training trials per day. During each trial, mice were placed into the tank at one of four designated start points per day in a pseudorandom order. Mice were trained for as many days as needed to reach the training criteria of 25 s (escape latency). If the mice failed to find the platform within 60 s, they were manually guided to the platform and allowed to remain there for 5 s. The probe trial was assessed 24 h after the last training session and consisted of a 60-s free swim in the pool without the platform. Performance was monitored with the EthoVision XT video tracking system (Noldus Information Technology, Leesburg, VA).

#### Contextual Fear Conditioning

During training, mice were placed in the fear-conditioning chamber (Ugo Basile Fear Conditioning chambers and controller; 4 chambers each equipped with a Monochrome GigE camera) and allowed to explore for 2 min 30 s before receiving one electric foot shock (duration, 2 s; intensity, 0.5 mA). Animals were returned to the home cage 30 s after the foot shock. Twenty-four hours later, behavior in the conditioning chamber was video recorded during 5 min and subsequently analyzed for freezing, which was defined as the absence of all movement except for respiration. Mice freezing activity was analyzed with Noldus Ethovision XT with the fear conditioning module.

### Tissue Preparation

After euthanasia, the animals were perfused transcardially with 0.1 M phosphate-buffered saline (PBS, pH7.4). Next, the hippocampus was used to collect synaptosomes [59], to stain with Golgi solution [60], for MSD analysis and for immunohistological stain.

### Golgi Stain

Mice were perfused transcardially with 0.1 M phosphate-buffered saline (PBS, pH 7.4), and brains were processed using superGolgi Kit (Bioenno Tech LLC, Santa Ana, CA), as described [60]. Brains were incubated for 11 days in impregnation solutions, followed by 2-day incubation in a post-impregnation solution. Once the impregnation of neurons was complete, thick (150 μm) free-floating sections were obtained using a HA752 vibratome (Campden Instruments Ltd, Lafayette, IN) and serially collected in a mounting buffer. Sections mounted on coated slides were stained and post-stained respectively for 20 min, dehydrated in graded ethanol, cleared with xylene, and coverslipped with DPX (VWR, Visalia, CA, USA) mounting medium.

### Dendritic Spine Analysis

Stereological quantifications were performed using Neurolucida software from Microbrightfield Bioscience (MBF Bioscience, Williston, VT, USA) to determine the number of spines in the stratum radiatum (sr) of the hippocampal CA1 region. Briefly, every second section was used through the entire antero-posterior extent of the hippocampus (between −1.46 mm anterior and −3.40 mm posterior to Bregma according to the atlas of Franklin and Paxinos, Third Edition, 2007). In the CA1 region, sr region was defined using a 5 × objective and spines were counted using a 100 × /1.4 objective (5–6 sections per animal, *n* = 5 animals per group).

### Synaptosome Extracts

Synaptosome extracts were prepared as described previously [59]. Briefly, the hippocampus was homogenized (using a Dounce homogenizer) in DEPC-treated water (Ambion) supplemented with 0.32 M sucrose, 20 mM Tris–HCl, 0.5 M EDTA, and 0.5 M EGTA (pH 7.4), containing complete protease (Sigma) and phosphatase inhibitor cocktails (Sigma). After homogenization, the crude synaptosomal fraction (synaptosomes plus mitochondria) was isolated by two sequential centrifugations (1,500 × *g*, 10 min followed by 12,500 × *g*, 20 min; at 4 °C). The protein content of the synaptosomal fractions was determined using the Bradford assay. For western blot experiments, synaptosomal preparations were temporally stored at −80 °C.

### Immunoblotting

Equal amounts of protein (20 μg) were separated on 10% Bis–Tris gel (Invitrogen, Carlsbad, CA) and transferred to nitrocellulose membranes. Membranes were blocked for 1 h in 5% (w/v) suspension of Bovine Serum Albumin (BSA; Gemini Bio-Products, West Sacramento, CA, USA) in 0.2% Tween 20 Tris-buffered saline (pH 7.5). After blocking, the membranes were incubated overnight at 4 °C with one of the following primary antibodies: anti-Drebrin (1:1000; Enzo Life Sciences), anti-GluA1 (1:1000; Cell Signaling), anti-*p*-GluA1 (Ser845; 1:1000; Cell Signaling), anti-postsynaptic density protein 95 (PSD95; 1:1000; Cell Signaling), anti-synaptic vesicle glycoprotein 2 (SV2A; 1:1000; Abcam), anti-fascin (1:1000; Abcam), anti-*p*-fascin (1:1000; Abcam), anti-synaptophysin (1:2000; Abcam), anti-β-tubulin (1:5000; Cell Signaling). The membranes were washed in tween-TBS for 20 min and incubated at 20 °C with the specific secondary antibody at a dilution of 1:10,000 (Pierce Biotechnology) for 60 min. The blots were developed using Super Signal (ThermoFisher Scientific, Rockford, IL, USA).

### Immunohistochemistry

Coronal free-floating Sects. (40 μm thick) were pretreated with 3% H2O2/3% methanol in Tris-buffered saline (TBS) for 30 min to block endogenous peroxidase activity. After TBS wash, sections were incubated first in TBS with 0.1% Trition X-100 (TBST) for 15 min, and then in TBST with 2% bovine serum albumin (BSA, Sigma-Aldrich) for 30 min. Sections were incubated with 6E10 (1:1000; BioLegend, San Diego, CA, USA), anti-HT7 (1:500; Thermo Scientific), and anti-AT180 (1:500; Thermo Scientific), in TBS + 5% normal horse serum overnight at room temperature. Sections were then incubated with biotinylated anti-mouse, 1:500 in TBS + 2%BSA + 5% normal serum for 1 h at 20 °C, followed by Vector ABC Kit and DAB reagents (Vector Laboratories, Burlingame, CA, USA) to visualize staining.

For double fluorescent stain, sections were incubated with anti-PSD95 (1:250; Invitrogen) and anti-synaptophysin (1:700; Sigma) overnight at 4 °C. Next, sections were incubated in secondary goat anti-mouse alexa-fluor 555 for synaptophysin and goat anti-rabbit alexa-fluor 488 for PSD95 antibody (Invitrogen) for 1 h. Sections were then mounted and coverslipped with Fluoromount-G (Southern Biothech).

### Quantitative Analyses

The biochemical data were quantitatively analyzed using Image J 1.36b software. For synaptophysin/PSD95 quantification, fluorescent sections were imaged with a Leica DM 2500 laser scanning confocal with identical laser and detection settings. Using a 63 × oil objective (zoom = 4), Z stacks (0.34 μm interval, within a depth of 3 μm) for each Sect. (6 sections per animal, *n* = 5) were collected per area of interest (CA1, stratum radiatum). Number of synaptic puncta and colocalization between PSD95 and synaptophysin puncta (number of colocalized spots at a distance of 200 nm or less) were quantified using Bitplane Imaris software (spots function).

### Aβ and Tau ELISA

Levels of Aβ and Tau were measured using the V-Plex Aβ peptide panel 1 kit and phospho-Thr231/total tau kit, respectively. For Aβ ELISA, 150 μl/well of Diluent 35 was added in a plate and incubated for 1 h at room temperature. After the washes, 25 μl of detection antibody solution and 25 μl of samples (soluble fraction, S1), calibrators or controls, were added to each well and incubated for 24 h at 4 °C. Then, samples were washed, 150 μl of Read buffer was added to each well, and the plate was read in the MESO QuickPlex SQ 120 instrument. For tau ELISA, 150 μl/well of Blocker A solution was first added and incubated for 1 h at room temperature. Then, the plate was washed and 25 μl of calibrator or samples was added in each well. Next, the plate was washed and incubated in the detection antibody solution (25 μl) for 1 h at room temperature. Finally, samples were washed, added 150 μl of Read buffer, and read in the MESO QuickPlex SQ 120 instrument. The data obtained were normalized with the protein concentration of each sample.

### In Vitro* Assay*

Primary neurons cultured from hippocampi of newborn rats were utilized to evaluate the effect of SPG302 on dendritic spine density in vitro. Briefly, neurons from the hippocampus of 3 newborn rats were cultured and plated in a 24-well plate and were cultured for 14 days. At day 1, the culture medium was replaced with plain medium. At day 4, 5 nM of araC was added in order to stop glial cell mitosis and to avoid background staining, multi-dimensional plains of focus, and excessive nutrient consumption. At day 14, SPG302 was added to the culture wells in several concentrations (0.1, 0.3, 1, 3, and 10 μM) using DMSO as a solvent. At day 15, cells were fixed on coverslips using methanol and double-stained with antibody against PSD95 for synaptic count of spines on the basal dendrites and antibody against synaptobrevin II for presynaptic imaging. DAPI staining was used for cell nuclei count. The number of spines per 20 μm length of dendrite was compared to the untreated control group. Vehicle group was treated with 0.1% DMSO.

For immunocytochemistry, coverslips with primary rat hippocampal neurons at day 15 were fixed with 100% frozen methanol and permeabilized with 0.02% Triton X-100. Neurons were then incubated in BSA for 1 h at room temperature followed by incubation with respective antibodies: mouse-anti-PSD95 (Thermofisher) and rabbit-anti-Synaptobrevin II (SYSY), and later on secondary antibodies Alexa Fluor 488-conjugated anti mouse antibody and Alexa Fluor 594-conjugated anti rabbit antibody. Coverslips were mounted on slides using fluorescent mounting medium with DAPI (Gbi). Approximately 10 images from different areas of a total of 3 coverslips per treatment group were taken. Imaging was done using BX43 Olympus microscope driven by the standard “CellSens” software by Olympus. Images were taken under 60 × water-dipping objective using DP74 camera (Olympus). Dendritic spines were detected automatically using ImageJ software. To perform analysis on the PSD95 content puncta, an exclusion threshold was set at 5 points above the background. Irrelevant content, such as cell bodies, glia and synapses out of the focus plain were dimmed. To estimate the neurites length, an ImageJ plugin – “NeuronJ” was used.

### Statistical Analyses

All data were analyzed by one-way or two-way analysis of variance (ANOVA), followed by Tukey’s comparisons using Graphpad Prism 8® software (Graphpad Prism Inc., San Diego, CA, USA). The significance was set at 95% of confidence. All values are presented as mean ± SEM.

## Results

### SPG302 Treatment Mitigates the Behavioral Impairments in 3xTg-AD Mice

**Fig. 1:**
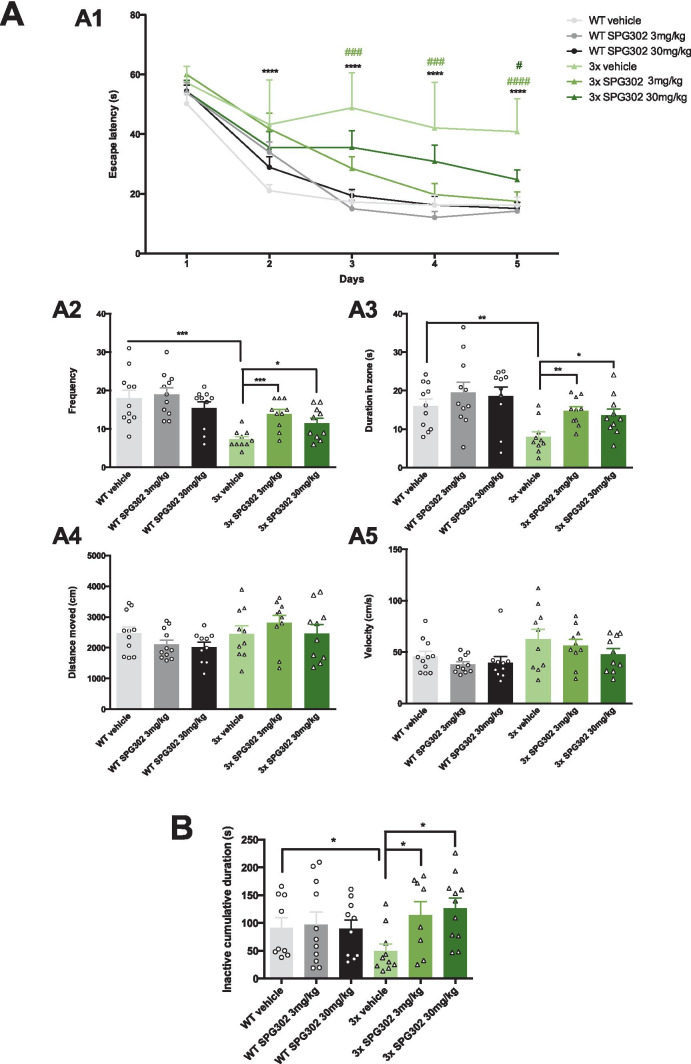
SPG302 restores behavioral deficits in 3xTg-AD mice. (**A**) Mice were trained on the spatial reference version of the MWM at 7 months of age. Acquisition curves (**A1**) are shown for the 5 days of training on the MWM. Two-way ANOVA: trials [F(4,285) = 92.63, *p* < 0.0001], treatment [F(5,285) = 30.33, *p* < 0.0001], and interaction [F(20,285) = 2.699, *p* = 0.0002], Tukey’s multiple comparison test, ****p < 0.0001, ***p < 0.001, *p < 0.05 (significance indicated for 3 × vehicle: * versus WT vehicle, # versus 3 × SPG302 3 mg/kg or 30 mg/kg). (**A2**) Frequency of WT vehicle, WT SPG302 3 mg/kg, WT SPG302 30 mg/kg, 3 × vehicle, 3 × SPG302 3 mg/kg and 3 × SPG302 30 mg/kg were measured 24 h after the last training session. Frequency is reduced in 3 × vehicle compared to WT vehicle. However, SPG302 treatment rescue frequency values in 3xTg-AD mice to WT mice. One-way ANOVA, ****p < 0.0001, F(5,56) = 7.828, Tukey’s multiple comparisons test, ***p < 0.001, *p < 0.05. (**A3**) Duration in zone. Time spent in the platform quadrant is reduced in 3x-vehicle vs WT-vehicle group. SPG302 treatment in 3xTg-AD mice increase the duration in zone time, similar to WT mice group. One-way ANOVA, **p = 0.0017, F(5,56) = 4.458, Tukey’s multiple comparisons test, **p < 0.01, *p < 0.05. Distance moved (**A4**) and velocity (**A5**) showed no differences between groups. (**B**) CFC analysis. 3 × vehicle showed a reduction in the inactivity duration which was recovered with SPG302 treatment to WT levels. *t*-test, *p < 0.05. *n* = 8–11 per group. The values represent means ± SEM

To evaluate the cognitive effects of SPG302, wild type (WT) and 3xTg-AD mice at 6 months of age were dosed daily for 4 weeks with either vehicle or SPG302 at 3 or 30 mg/kg (i.p.), with behavioral assessments conducted during the 4th week, followed by sacrifice for performance of histological and biochemical assays in brains from the same animals. To assess whether SPG302 rescues cognitive function in 3xTg-AD mice, we evaluated spatial memory in vehicle and SPG302-treated WT and 3xTg-AD mice using the Morris water maze (MWM) test (Fig. 1A). 3xTg-AD mice treated with vehicle showed severe impairments in learning during acquisition of the spatial task compared to vehicle-treated WT mice. These deficits were reversed by SPG302 treatment at both 3- and 30-mg/kg doses (Fig. 1A1). In addition, mice were tested 24 h after the last training trial to evaluate memory retention. Vehicle-treated 3xTg-AD mice displayed significant impairments in retention as measured by the frequency of visits to, and time spent in, the target area (Fig. 1A2-A3). Treatment of 3xTg-AD mice with SPG302 at 3 and 30 mg/kg significantly improved memory retention in these assays. Moreover, the cognitive impairments observed in 3xTg-AD vehicle mice were not attributed to motor deficits, since no significant changes were noted for distance traveled and velocity (Fig. 1A4-A5) in any of the treatment groups (including WT and 3xTg-AD mice).

To further characterize the memory enhancing effects of SPG302 in 3xTg-AD mice, we used contextual fear conditioning (CFC) paradigm, another hippocampal-dependent task (Fig. 1B). Vehicle-treated 3xTg-AD mice showed a markedly reduced freezing response to the conditioning context compared to WT vehicle mice. The duration of the freezing response was significantly increased after SPG302 treatment in 3xTg-AD mice and was similar to WT levels. Overall, these data suggest that SPG302 reverses hippocampal learning and memory deficits in 3xTg-AD mice.

### SPG302 Restores the Numbers of Postsynaptic Puncta and Their Colocalization with Presynaptic Elements in 3xTg-AD Mice

**Fig. 2:**
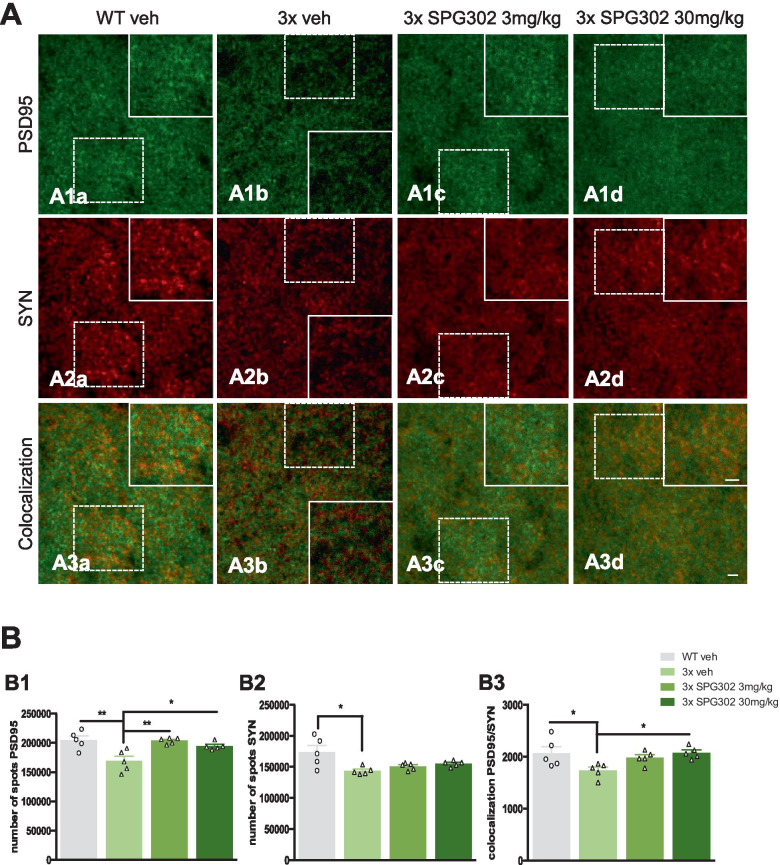
SPG302 treatment rescue synaptic puncta density in 3xTg-AD mice. (**A**) Confocal images of PSD95 and synaptophysin in the stratum radiatum of CA1 hippocampal area. (**B**) Quantitative analysis of these images revealed a reduction in the number of PSD95 (**B1**) (one-way ANOVA, **p = 0.0013, F(3,16) = 8.537, Tukey’s multiple comparisons tests, **p < 0.01, *p < 0.05) and SYN (**B2**) (one-way ANOVA, *p = 0.0134, F(3,16) = 4.894, Tukey’s multiple comparisons tests, *p < 0.05) puncta as well as the colocalization between both markers (**B3**) (one-way ANOVA, *p = 0.0274, F(3,16) = 3.960, Tukey’s multiple comparisons tests, *p < 0.05) in 3 × vehicle compared to WT vehicle. SPG302 treatment restores PSD95 number of spots and colocalization. However, no changes were observed with the treatment for the presynaptic marker synaptophysin. *n* = 5 per group. The values represent means ± SEM. Scale bars: 50 μm

We next evaluated whether the SPG302-induced improvement of 3xTg-AD mice in hippocampal-dependent memory tasks was associated with changes in pre- and postsynaptic protein puncta in the stratum radiatum of hippocampal field CA1 using immunofluorescence microscopy. Analysis of sections immunolabeled for the postsynaptic scaffolding protein, PSD95, revealed a significant deficit in the number of postsynaptic puncta (Fig. 2A1a-1d, and B1) in 3xTg-AD mice treated with vehicle (Fig. 2A1b) compared to WT vehicle mice (Fig. 2A1a), which was restored by SPG302 treatment at both 3 and 30 mg/kg doses (Fig. 2A1c, 1d). Decreases in the number of presynaptic elements containing synaptophysin in 3xTg-AD mice were also observed, but this was not rescued by SPG302 treatment at 3 or 30 mg/kg (Fig. 2A2a-2d and B2). However, SPG302 treatment (at 30 mg/kg) rescued deficits in the colocalization of PSD95- and synaptophysin-labeled elements (Fig. 2A3a-3d) in 3xTg-AD mice at 30 mg/kg mice (Fig. 2B3), considered an immunohistochemical measure of possible functional synapses.

**Fig. 3:**
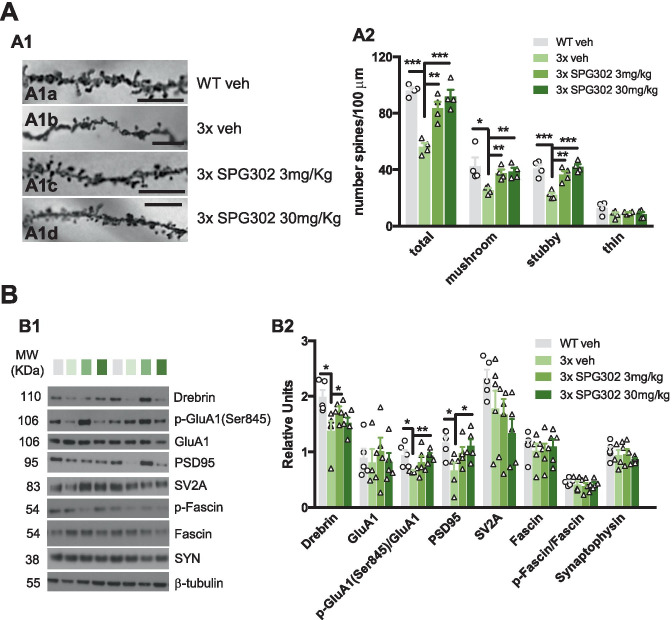
SPG302 improve dendritic spine density in 3xTg-AD mice and synaptic-related proteins. (**A**) Dendritic spines analysis in WT vehicle, 3 × vehicle, 3 × SPG302 3 mg/kg and 3 × SPG302 30 mg/kg. Light microscopic images of radiatum layer in CA1 subfield (**A1**) in WT vehicle (**A1a**), 3 × vehicle (**A1b**), 3 × SPG302 3 mg/kg (**A1c**) and 3 × SPG302 30 mg/kg (**A1d**). (**A2**) Stereological quantification showed a significant decrease in the density of total dendritic spines in 3x-vehicle group compared to WT-vehicle group (one-way ANOVA, ****p < 0.0001, F(3,12) = 23.28, Tukey’s multiple comparisons tests, ***p < 0.001, **p < 0.01), in mushroom (one-way ANOVA, *p = 0.0249, F(3,12) = 4.482, Tukey’s multiple comparisons tests, **p < 0.01, *p < 0.05) and stubby spines (one-way ANOVA, ***p < 0.0001, F(3,12) = 16.80, Tukey’s multiple comparisons tests, ***p < 0.001, **p < 0.01). Notably, SPG302 treatment in 3xTg-AD mice rescue total, mushroom and stubby spines compared to vehicle (*n* = 4 per group). (**B**) Immunoblot analysis of Drebrin, GluA1, p-GluaA1, PSD95, SV2A, Fascin, p-Fascin and synaptophysin in hippocampal synaptosome from WT vehicle, 3 × vehicle, 3 × SPG302 3 mg/kg and 3 × SPG302 30 mg/kg group are shown in alternate lanes (**B1**). (**B2**) Quantification normalized to β-tubulin for Drebrin, GluA1, PSD95, SV2A, Fascin and synaptophysin, normalized to GluA1 for p-GluA1, and to Fascin for p-Fascin, and expressed as relative units, showed a significant reduction in Drebrin, p-GluA1 and PSD95 in 3 × vehicle vs WT vehicle group. Notably, a significant increase in Drebrin (3 × SPG302 3 mg/kg), p-GluA1 (3 × SPG302 30 mg/kg) and PSD5 (3 × SPG302 30 mg/kg) is shown in 3 × treated with SPG302 compared to 3x-vehicle group. Unpaired *t* test, **p < 0.01, *p < 0.05. *n* = 5 per group. The values represent means ± SEM. Scale bars: 6.25 μm

### SPG302 Reverses Large Decreases in Dendritic Spine Density in 3xTg-AD Mice and Enhances Spinogenesis In Vitro

SGP302 and its prototype compounds promote the formation of new dendritic spine synapses through a mechanism of action that targets the F-actin-based cytoskeleton. We thus sought to validate that SPG302 increases dendritic spine density in the 3xTg-AD model as expected and as suggested by both the behavioral immunohistochemical data. We performed Golgi staining and stereological quantification of dendritic spines in the stratum radiatum (sr) of hippocampal field CA1 (Fig. 3A1, A2). The stereological quantification indicated that 3xTg-AD mice treated with vehicle have significantly reduced dendritic spine density (Fig. 3A1, A2) compared to vehicle-treated WT mice, particularly in mushroom and stubby-type spine profiles. These large deficits in dendritic spines in 3xTg-AD mice (~ 35–50%) were reversed by treatment with SPG302 at 3 and 30 mg/kg (Fig. 3A1, A2), which increased spine density in 3xTg-AD mice to levels not statistically different from those seen in vehicle-treated WT mice.

We also evaluated the effect of SPG302 in vitro on the density of dendritic spines on primary hippocampal neurons from rat (Supplementary Fig. 1A). Treatment with SPG302 at concentrations of 0.1, 0.3, 1, 3, and 10 μM resulted in a statistically significantly increase in the mean spine density (spines/20 μm) compared to the vehicle group (37.41 ± 5.88, 33.72 ± 2.75, 35.65 ± 3.68, 45.97 ± 3.89, 24.71 ± 2.33, respectively, vs. 12.54 ± 1.81 in vehicle controls). Treatment with vehicle (0.1% DMSO) did not increase the mean spine density compared to an untreated control group (14.76 ± 1.02 vs. 12.54 ± 1.81, respectively).

To further understand the nature of SPG302’s synaptic rescue effects, we measured the levels of several key synaptic proteins including Drebrin, GluA1, p-GluA1, PSD95, synaptic vesicle glycoprotein A (SV2A), synaptophysin and the molecular target of SPG302, and synaptophysin, by western blot (WB) in hippocampal synaptosomes (Fig. 3B1) prepared from a subset of animals tested in behavioral assays. Changes in the levels and activity of these synaptic markers have been associated with memory impairments in AD [38, 57, 61, 62]. In accord with the observed spine deficits 3xTg-AD mice, WB analysis revealed significant decreases in the steady-state levels of Drebrin, PSD95, and the p-GluA1/GluA1 ratio in 3xTg-AD vehicle mice compared to WT vehicle mice. The levels of these proteins were significantly improved with SPG302 treatment (Fig. 3B1, B2). These data suggest that the structural effects of SPG302 on dendritic spine density are accompanied by increases in key scaffolding, actin regulatory, and glutamatergic-signaling proteins involved in the formation and plasticity of glutamatergic synapses.

### SPG302 Does Not Alter Aβ or Tau Pathology in 3xTg-AD Mice

**Fig. 4:**
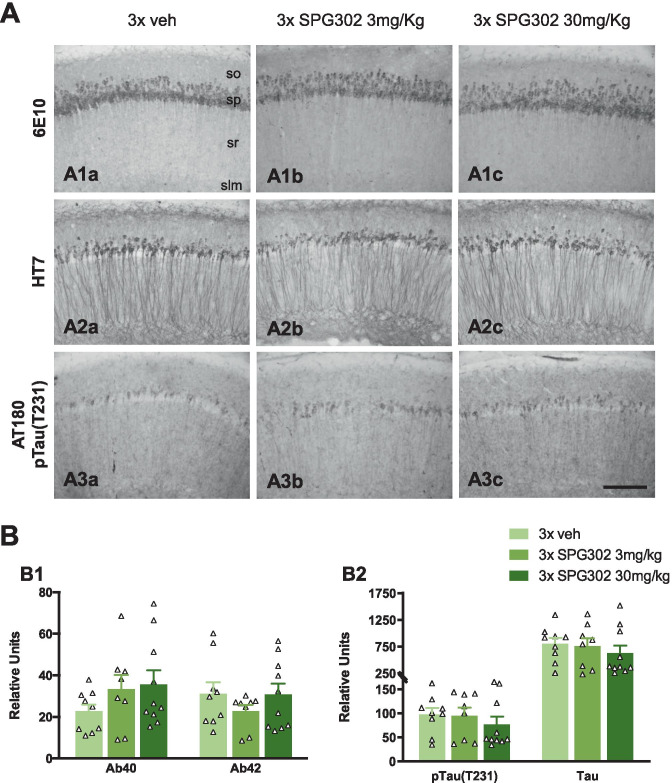
SPG302 treatment does not alter Aβ or tau pathology. (**A**) Light microscopic images stained with 6E10 (**A1a**-**A1c**), HT7 (**A2a**-**A2c**) and AT180 (**A3a**-**A3c**) in 3 × vehicle (**A1a**, **A2a** and **A3a**), 3 × SPG302 3 mg/kg (**A1b**, **A2b** and **A3b**) and SPG302 30 mg/kg (**A1c**, **A2c** and **A3c**). No differences were detected in the staining of these three markers between the different groups. (**B**) Amyloid (**B1**), pTau (**B2**) and Tau (**B2**) levels measured by ELISA in 3 × vehicle, 3 × SPG302 3 mg/kg and 3 × SPG302 30 mg/kg revealed no differences for any of these markers between the groups. *n* = 8–10 per group. The values represent means ± SEM. so, stratum oriens; sp, stratum pyramidale; sr, stratum radiatum; slm, stratum lacunosum-moleculare. Scale bars: 100 μm

Accumulation of Aβ and hyperphosphorylated Tau are the hallmark features of AD molecular pathology [63], so any drug affecting AD progression — even if not targeting these pathways directly — may alter one or both of these characteristics. However, Aβ pathology was not altered by SPG302 treatment in 3xTg-AD mice as measured by immunohistochemistry (Fig. 4A1a-A1c) and by ELISA (Fig. 4B1). Moreover, immunohistochemical and ELISA analysis revealed that neither steady-state Tau (HT7) nor phospho-Tau species recognized by AT180 (Thr231) were altered by SPG302 treatment (Fig. 4A2a-A3c and B2). The data suggest that the effect of SPG302 in restoring synaptic and cognitive deficits in 3xTg-AD was not associated with any change in Aβ and/or Tau pathology.

## Discussion

Synapse loss is an early and nodal event in the pathogenesis of AD that correlates with cognitive decline better than the hallmark features of AD brain, amyloid plaques, and neurofibrillary tangles [16, 19, 20]. However, while the rationale for considering synaptic regeneration as an endpoint in AD is very strong, the candidate therapeutics and data that can be directed toward validating this hypothesis are sparse at best [38]. In this study, we demonstrated that a novel spinogenic small molecule, SPG302, is capable of reversing deficits in cognition and synaptic density in a mouse model of AD, effects that were accompanied by increases in the expression of key postsynaptic proteins and the colocalization of pre- and post-synaptic markers. The efficacy of SPG302 is thus of high translational significance because it provides a clear and robust preclinical demonstration that promoting the regeneration of glutamatergic synapses is a viable therapeutic approach in AD. It also suggests that ABPs and other regulators of the synaptic F-actin-based cytoskeleton offer viable targets to achieve this end.

Several aspects of the effects of SPG302 on spine density inform on its potential as a therapeutic at the clinical stage. First, it is notable that spine density was returned to levels not statistically different from WT controls. Moreover, the spines induced included both stubby and mushroom-shaped contacts that are preferentially lost in human AD brain and many AD mouse models [37, 64]. These observations suggest that SPG302 induces physiologically relevant spinogenesis, and that new synapses attain a normal distribution of shapes and, to the extent spine shape and synaptic strength are linked [65], efficacy states. This is remarkable given that there is no a priori reason to expect that the effects of any synaptogenic therapeutic agent will be constrained to those that are physiologically relevant, particularly against a background of AD-like pathology. We hypothesize that some instructive cue(s) may remain in the local dendritic microenvironment — perhaps in the extracellular matrix, the proximity of axons or their boutons, or local variations in glutamate concentration — that dictate where and how many new synapses should be formed, thus constraining SPG302’s effects within physiologically relevant limits. A potentially informative observation in this regard comes from hibernation. It has been shown that synaptic density decreases during torpor coincident with an AD-like hyperphosphorylation and redistribution of Tau (presumably to prevent excitotoxicity in the context of the severe metabolic stresses of hibernation). Shortly after arousal and return to euthermia, both synaptic density and Tau phosphorylation and distribution normalize to normal levels [66–69]. Moreover, memory traces laid down before hibernation remain intact [70, 71], suggesting that synapses are regenerated at physiologically relevant densities and efficacy states based on the persistence of some endogenous instructive cue. Second, the question arises as to whether SPG302-induced spinogenesis simply outpaces synaptic loss, which is already severe at 6mo in the 3xTg-AD mouse, or if the effects of the drug create a new stable state that is disease modifying at some level of AD pathogenesis. The present data do not answer this question, but it is reasonable to consider that normalization of synaptic density, particularly the density of large, high-efficacy mushroom-shaped spines, may revitalize trophic support and other mechanisms that aid in the maintenance of synapses and are disease modifying.

Another quality of SPG302’s effects on spine density that informs on its potential utility as a therapeutic is its rapidity of action. In the present study, we observed rescue of synaptic and cognitive deficits within 1 month of dosing. Prototypes of SPG302 were effective at restoring motor performance in a rodent model of traumatic brain injury within 1 week of dosing [72], and in vitro they induce spinogenesis within an hour or less [73]. These data suggest that SPG302, and potentially other spinogenic small molecules, may be efficacious in humans on a much shorter time scale than has been expected of other candidate therapeutics that have been tested in AD. For example, treatment durations of 18 months or more were expected to be necessary to observe significant effects of Aβ-targeted monoclonal antibodies such as aducanumab [74], and this was only in terms of slowing disease progression, not reversing cognitive decline. While the molecular target of SPG302 is not here revealed, it is notable that its activity on the F-actin cytoskeleton is conceptually consistent with its rapid effects on spines and cognition. The formation and maturation of new spines, which depend on the formation of branched F-actin assemblies, can be triggered by very brief inductive events. For instance, in the developing hippocampus, induction of LTP with activity patterns that last on order of seconds can lead to spinogenesis that develops over the course of hours [75]. Based on the function of SPG302’s target (for which SPG302 is highly selective), our working hypothesis is that SPG302 triggers the rapid formation of branched F-actin assemblies that are an essential basis of spine formation and maturation [45, 46]. Clinically, this may mean that even brief exposure to SPG302 on a daily basis may be sufficient to produce rapid therapeutic effects in AD and other synaptopathies.

Though we did not have a specific prediction in this study as to the potential effects of SPG302 on levels of Aβ and phospho-Tau, we nevertheless measured these hallmark features of AD molecular pathogenesis. SPG302 treatment did not alter the levels of Aβ or phospho-Tau in the hippocampus of 3xTg-AD mice relative to vehicle controls. The dissociation between phenotypic rescue and Aβ load is not unprecedented. Rescue of synaptic density and cognition with no apparent effects on Aβ load, or even an increase in Aβ, have been observed in preclinical studies that assessed the effects of modulating progranulin, microglial, and other factors in AD models [76, 77]. Such findings accord with data from human studies showing that some subjects with robust Aβ load can be cognitively normal. Spine density, particularly stubby and mushroom profiles, may be a key determinant of this dissociation insofar as subjects who are cognitively normal despite having high Aβ load have more dendritic spines [37]. The lack of an effect of SPG302 on levels of phospho-Tau may pose a different question. The levels and regional extent of neurofibrillary tangles correlate with clinical status much better than Aβ load. Moreover, there is compelling evidence that Tau pathology is a proximal cause of synaptic failure. This raises the question of how SPG302 can restore synaptic density and cognition in the context of high levels of phospho-Tau, particularly when the animal model used expresses the P301L mutation that is known to induce synapse loss in other tauopathy models as well [78]. While this will require more study, it is possible that cues for physiologically relevant densities and patterns of new synapse formation remain intact in the face of AD-like Tau pathology. This possibility finds support in observations that high levels of phospho-Tau occur in the brains of hibernating animals during torpor, when synaptic density is reduced, but are quickly diminished upon return to euthermia [66, 67]. Insofar as synaptic density rebounds and memories remain, it is reasonable to assume that some cue for synaptogenesis — perhaps even a residual synaptic contact — survives robust Tau pathology. In AD, 3-dimensional electron microscopy studies of synapses in the transentorhinal cortex suggest that at least some of the reduction in dendritic spines may be due to a conversion to shaft synapses [33].

Consistent with the formation of new axospinous synaptic contacts by SPG302, we observed increases in key synaptic proteins and a higher colocalization of pre- and post-synaptic markers. Importantly, synapse-like puncta immunopositive for the postsynaptic scaffolding protein, PSD95, were significantly increased by SPG302 treatment, as was the number of PSD95-positive elements colocalized with the presynaptic marker synaptophysin (at 30 mg/kg). Prior work has shown that the synaptic content of PSD95 scales positively with spine size and synaptic efficacy [65, 79]. In addition, SPG302 significantly increased the levels of both PSD95 (at 30 mg/kg) and Drebrin A (at 3 mg/kg) in synaptic fractions. Drebrin A is a postsynaptic ABP that promotes the enlargement and stabilization of synapses. Levels of Drebrin A, and spines containing the protein, decline early in AD and animal models [32, 48, 49]; its upregulation by SPG302 is consistent with the formation of larger, mushroom-shaped spines and suggests a normalization of synaptic cytoskeletal function by the drug. The above effects of SPG302, along with our Golgi staining and behavioral data (highly significant at both doses), indicate that SPG302 induced the formation of new synapses. Accordingly, we also observed increased levels of phospho-GluA1 in synaptic fractions. GluA1 is a subunit of amino-3-hydroxy-5-methyl-4-isoxazolepropionic acid receptors (AMPARs), which mediate fast excitatory transmission and are impacted in AD [57, 80]. Our lab and others have shown that AMPA signaling is compromised early in the disease course in an AD transgenic mouse model [57, 81]. AMPARs are composed of four different subunits (GluA1-4, or Gria1-4). Of these, the role of the GluA1 subunit in learning and memory processes is the most widely investigated [82–84], including its phosphoregulation at several sites, including Ser831 and Ser845 [85]. Notably, a decline in AMPA signaling is associated with changes in the structure and number of dendritic spines in an AD model [57]. Rescue of phospho-GluA1 levels in synaptic fractions is yet another indication that SPG302 treatment increased the levels of axospinous glutamatergic contacts in the 3xTg-AD mouse model.

In sum, our data provide compelling preclinical evidence that SPG302 and related spinogenic small molecules may be of high therapeutic value in AD, as well as other synaptopathies. At present, there are no FDA-approved therapies that address synapse loss in any of the synaptopathies, making SPG302 a highly differentiated candidate therapy for these disorders. Among the small set of therapeutics that may be able to regenerate lost synapses [38], SPG302 remains highly unique. The molecular target of SPG302 has not yet been the subject of clinical development in CNS conditions, and its mechanism of action may be superior to these other approaches. Moreover, it has excellent drug-like properties. A notable comparison is a class of HDAC inhibitors being developed for frontotemporal dementia and other neurodegenerative conditions [86]. These compounds increase the density of spine synapses in animal models, but only that of thin spines; thus, they do not restore the normal spectrum of synaptic shapes that includes mushroom and stubby spines. In principle, SPG302 could be used as a monotherapy or as an add-on to other therapeutics in development that target upstream aspects of AD pathogenesis at the molecular level. Regarding the latter, it is important to consider that AD involves more than degeneration — it is also a failure of regenerative processes that can compensate for synaptic failure.

## Conclusions

New disease-modifying therapeutics that target degenerative mechanisms may halt AD progression, but fail to elicit gains in cognition that actually improve the quality of life and reduce the economic and emotional burden AD imposes on affected individuals and their families. Should SPG302 prove safe and well tolerated in humans, the rapid regenerative effects of SPG302 that we report here raise the prospect of a much different clinical experience than has been the case so far for AD and other synaptopathies in terms of duration of treatment, outcome measures, and expected effect sizes.

## Supplementary Information

Below is the link to the electronic supplementary material.

Supplementary file1 (MP4 171645 kb)

Supplementary file2 (MP4 195702 kb)

Supplementary file3 (MP4 164887 kb)

Supplementary file4 (PDF 508 kb)

Supplementary file5 (PDF 525 kb)

Supplementary file6 (PDF 533 kb)

Supplementary file7 (PDF 524 kb)

Supplementary file8 (PDF 257 kb)

Supplementary file9 (PDF 550 kb)

Supplementary file10 (PDF 533 kb)

Supplementary file11 (PDF 542 kb)

Supplementary file12 (PDF 525 kb)

Supplementary file13 (PDF 533 kb)

Supplementary file14 (PDF 525 kb)

Supplementary file15 (PDF 524 kb)

Supplementary file16 (PDF 516 kb)

Supplementary file17 (DOCX 12 kb)

## Abbreviations

3xTg-AD: Triple transgenic Alzheimer’s disease
Aβ: Amyloid-beta
ABPs: Actin-binding proteins
AChE: Acetyl cholinesterase
AD: Alzheimer’s disease
AMPAR: Amino-3-hydroxy-5-methyl-4-isoxazolepropionic acid receptor
ANOVA: Analysis of variance
BSA: Bovine serum albumin
CFC: Contextual fear conditioning
F-actin: Filamentous actin
KI: Knock in
LTD: Long-term depression
LTP: Long-term potentiation
MWM: Morris water maze
NMDAR: N-methyl-D-aspartate receptor
PBS: Phosphate buffered saline
PSD95: Postsynaptic density protein 95
sr: Stratum radiatum
SV2A: Synaptic vesicle glycoprotein 2
TBS: Tris-buffered saline
WB: Western blot
WT: Wild type
