# Supplementary material for: SPG302 Reverses Synaptic and Cognitive Deficits Without Altering Amyloid or Tau Pathology in a Transgenic Model of Alzheimer’s Disease
Source: Neurotherapeutics. 2021 Nov 4;18(4):2468–83. doi: 10.1007/s13311-021-01143-1 (PMC8804111; doi:10.1007/s13311-021-01143-1)
Supplement: Supplementary file 17 — Supplementary file17 (DOCX 12 kb) [file 13311_2021_1143_MOESM17_ESM.docx]

***Supplemental Figure 1. SPG302 stimulates dendritic spine formation in primary hippocampal neurons.*** A) Spines/20 μm in control neurons, neurons treated with vehicle and neurons treated with SPG302 (0.1, 0.3, 1, 3 and 10 μM). SPG302 treatment significantly increases the number of spines in hippocampal neurons (One-way ANOVA, ****p<0.0001, F(6,33)=12.91, Tukey’s multiple comparisons test, ****p<0.0001, ***p<0.001, **p<0.01).

***Supplemental Figure 2. Synaptic puncta quantification using Imaris software.*** These videos are an example of how the analysis of SYN (A), PSD95 (B) and PSD95/SYN colocalization (C) was performed. The hippocampal stratum radiatum images were collected using Z stacks and PSD95 and synaptophysin puncta were considered colocalized when the spots were at 200 nm or less apart.
